# Differentially expressed proteins in glioblastoma multiforme identified with a nanobody-based anti-proteome approach and confirmed by OncoFinder as possible tumor-class predictive biomarker candidates

**DOI:** 10.18632/oncotarget.17390

**Published:** 2017-04-24

**Authors:** Ivana Jovčevska, Neja Zupanec, Žiga Urlep, Andrej Vranič, Boštjan Matos, Clara Limbaeck Stokin, Serge Muyldermans, Michael P. Myers, Anton A. Buzdin, Ivan Petrov, Radovan Komel

**Affiliations:** ^1^ Medical Center for Molecular Biology, Institute of Biochemistry, Faculty of Medicine, University of Ljubljana, Ljubljana, Slovenia; ^2^ Center for Functional Genomics and Bio-Chips, Institute of Biochemistry, Faculty of Medicine, University of Ljubljana, Ljubljana, Slovenia; ^3^ Department of Neurosurgery, Foundation Rothschild, Paris, France; ^4^ Department of Neurosurgery, University Clinical Center, Ljubljana, Slovenia; ^5^ Institute of Histopathology, Charing Cross Hospital, London, United Kingdom; ^6^ Cellular and Molecular Immunology, Vrije Universiteit Brussel, Brussels, Belgium; ^7^ International Center for Genetic Engineering and Biotechnology, Trieste, Italy; ^8^ First Oncology Research and Advisory Center, Moscow, Russia; ^9^ National Research Center ‘Kurchatov Institute’, Center of Convergence of Nano-, Bio-, Information and Cognitive Sciences and Technologies, Moscow, Russia; ^10^ Center for Biogerontology and Regenerative Medicine, IC Skolkovo, Moscow, Russia; ^11^ Moscow Institute of Physics and Technology, Moscow, Russia

**Keywords:** glioblastoma multiforme, biomarkers, nanobodies, cancer biology, OncoFinder

## Abstract

Glioblastoma multiforme is the most frequent primary malignancy of the central nervous system. Despite remarkable progress towards an understanding of tumor biology, there is no efficient treatment and patient outcome remains poor. Here, we present a unique anti-proteomic approach for selection of nanobodies specific for overexpressed glioblastoma proteins. A phage-displayed nanobody library was enriched in protein extracts from NCH644 and NCH421K glioblastoma cell lines. Differential ELISA screenings revealed seven nanobodies that target the following antigens: the ACTB/NUCL complex, VIM, NAP1L1, TUFM, DPYSL2, CRMP1, and ALYREF. Western blots showed highest protein up-regulation for ALYREF, CRMP1, and VIM. Moreover, bioinformatic analysis with the OncoFinder software against the complete “Cancer Genome Atlas” brain tumor gene expression dataset suggests the involvement of different proteins in the WNT and ATM pathways, and in Aurora B, Sem3A, and E-cadherin signaling. We demonstrate the potential use of *NAP1L1*, *NUCL*, *CRMP1*, *ACTB*, and *VIM* for differentiation between glioblastoma and lower grade gliomas, with *DPYSL2* as a promising “glioma *versus* reference” biomarker. A small scale validation study confirmed significant changes in mRNA expression levels of *VIM*, *DPYSL2*, *ACTB* and *TRIM28*. This work helps to fill the information gap in this field by defining novel differences in biochemical profiles between gliomas and reference samples. Thus, selected genes can be used to distinguish glioblastoma from lower grade gliomas, and from reference samples. These findings should be valuable for glioblastoma patients once they are validated on a larger sample size.

## INTRODUCTION

Glioblastoma multiforme (GBM) is the most fre-quent and lethal form of primary brain tumor, with an annual incidence of 5.26 per 100,000 people, or 17,000 new cases diagnosed yearly worldwide [1]. GBM is more common among Caucasian men and is typical of advanced age. Clinical management consists of maximal surgical resection followed by radiation and chemotherapy [2–4]. As a result of its infiltrative growth, in the majority of cases, GBM generally reoccurs within 7 months to 10 months after surgical intervention [5–7]. Despite treatment, most patients succumb to the disease some 12 months to 18 months after diagnosis, while for recurrent GBM, life expectancy is ~6 months [1, 8–12]. Due to this short survival with currently available therapies, alternative treatments are under extensive exploration [2, 13–16].

Among the major issues in GBM management are rapid tumor growth, tumor location, and late diagnosis. The general late diagnosis of GBM is a consequence of the asymptomatic nature of the early disease stages, which present with nausea, headache, and cognition changes. To better define GBM, a number of proteins have been proposed as possible biomarkers, including the EGFR/EGFRvIII pair, and the glycoprotein CD133 [17–22]. Further studies have investigated patient serum and cerebrospinal fluid for possible biomarkers, although the specificities of all of these candidate proteins remain to be validated [23–26].

An alternative approach for discovering proteins with specificity for GBM is offered by using nanobodies, which are antigen-binding fragments that are derived from naturally occurring heavy-chain-only antibodies in camelids [27, 28]. Importantly, the complete antigen-binding fragment of a nanobody is encoded by a single gene fragment, the heavy-chain variable region, or VHH. Thus this avoids gene splitting and scrambling during cloning, which happens with single-chain variable fragments [29]. In addition, their extended H3 loop that is responsible for antigen binding predicts the specificity for novel epitopes that can be hidden from classical antibodies and conventional proteomic techniques. Furthermore, nanobodies possess exceptional and beneficial features including small size (~14 kDa), high resistance to non-physiological pH and elevated temperature, water solubility, resistance to aggregation, and simple and inexpensive production in bacteria.

This straightforward anti-proteomic approach led to the identification of seven candidate biomarkers that conventional techniques have failed to uncover. This finding illustrates that the nanobody technology is a suitable alternative approach for identification of GBM-specific proteins and it has provided a good starting point for further investigation of their roles. Potential use of *DPYSL2* as a “glioma *versus* reference” tissue biomarker, and the roles of some of the suggested biomarkers for class differentiation have been confirmed with qPCR on a small scale study, but need to be validated with a larger sample number.

## RESULTS

### Glioblastoma multiforme target-specific nanobodies

**Figure 1:**
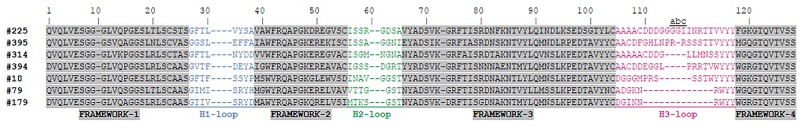
Nanobody sequences The selected nanobodies show the characteristic starting (QVQL or DVQL) and ending (TVSS) nanobody sequences. Different H3 loops imply that all of these nanobodies bind to different antigens; i.e., different proteins of interest. The presence of the GLEW sequence motif in the FR2 region of Nb10 indicates its *VH* germline origin during the *V-D-J* recombination, whilst the rest of the nanobodies definitely have a *VHH* germline origin, as e.g. for the FR2 sequences that have the VHH-typical Arg50. Amino acid sequences of the H3 loops are given in alphabetical order.

The nanobody library against GBM cells comprised 10^8^ individual transformants, which is consistent with the average size of a high quality immune nanobody library [30]. Phage enrichment during panning on protein extracts of GBM stem-like cell lines was good, as there were at least two-fold more bacteria infected with viral particles retrieved from GBM samples than from reference samples. After the second and third round of panning, large numbers of bacteria were grown and their periplasmic proteins were screened by ELISA. Proteins from the periplasm that showed at least 1.5-fold higher ELISA signals in wells with GBM lysate than in wells with reference lysate were considered positive. Numerous ELISA screenings led to the identification of seven nanobodies with specificity for GBM proteins: Nb10, Nb79, Nb179, Nb225, Nb314, Nb394, and Nb395, with GBM/ reference ELISA ratios of 1.54, 2.27, 1.68, 2.17, 2.25, 1.53, and 3.29, respectively. The nanobody genes obtained after Sanger sequencing were translated *in silico* to their amino acid sequence and revealed the characteristic starting (i.e., QVQL, DVQL) and ending (i.e., TVSS) amino acid sequences [31, 32]. A unique H3 region for each nanobody suggested that they might recognize different antigens (Figure 1).

### Antigens recognized by nanobodies

The purified nanobodies were used to immune-capture their cognate targets in protein lysates from GBM stem-like cell lines. Using a ≤5% false-discovery rate, the captured antigens were identified by mass spectrometry, as: Nb10: β-actin/nucleolin (ACTB/NUCL) complex; Nb79: vimentin (VIM); Nb179: nucleosome assembly protein 1 like (NAP1L1); Nb225: Tu translation elongation factor, mitochondrial (TUFM); Nb314: dihydropyrimidinase-related protein 2 (DPYSL2) and/or methylenetetrahydrofolate dehydrogenase 1 (MTHFD1); Nb394: collapsin response mediator protein 1 (CRMP1); and Nb395: ALY/REF export factor (ALYREF).

### Differential protein occurrence in glioblastoma, lower grade glioma, and reference samples

**Figure 2:**
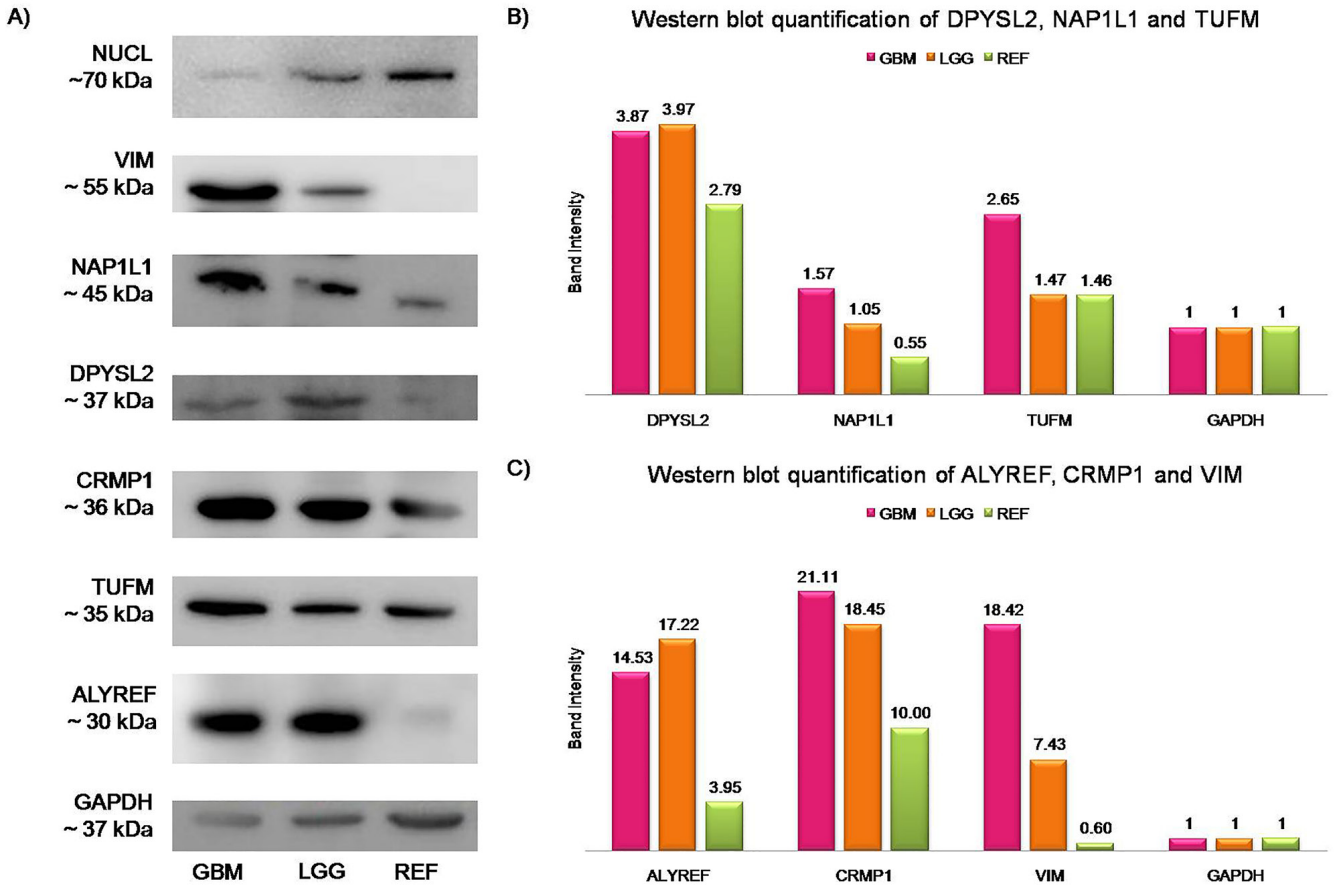
Western blotting validation and quantification of the identified proteins The band intensity of each protein was plotted after normalization to the GAPDH signal of the same lane, as *band intensity* =AU(antigen)AU(GAPDH). GAPDH was the loading control. (**A**) Representative Western blotting of the antigens identified. GBM, cytosolic protein extract from GBM tissues; LGG, cytosolic protein extract from lower grade gliomas; REF, cytosolic protein extract from reference samples. (**B**) Protein expression for DPYSL2, NAP1L1, and TUFM, with 1.38, 2.86, and 1.82 higher expression levels in GBM compared to reference samples, respectively. (**C**) Protein expression for ALYREF, CRMP1, and VIM, that exhibit up-regulation in GBM with 3.68-, 2.11-, and 30-fold increases compared to the reference samples, respectively.

**Figure 3:**
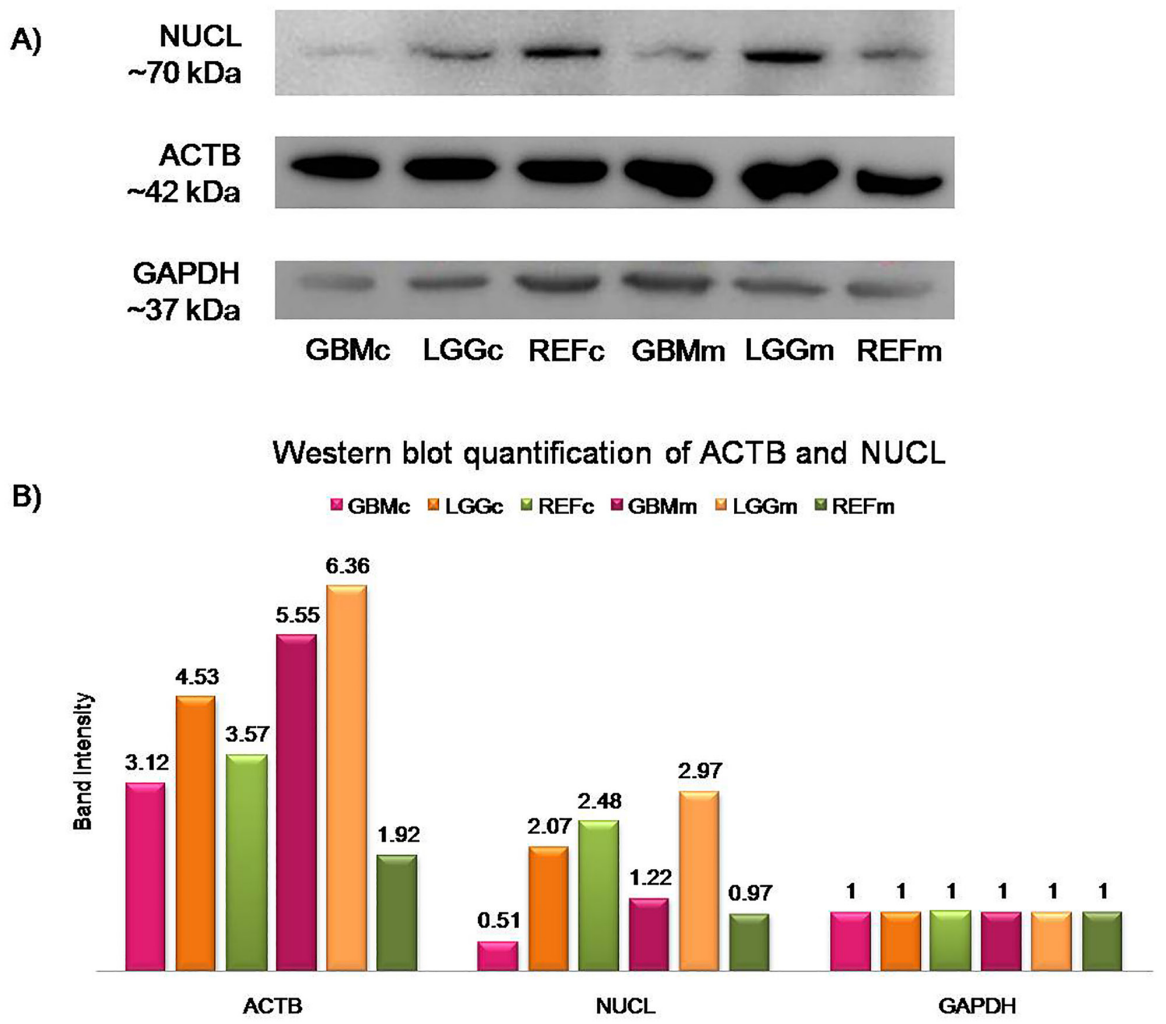
Quantification of NUCL and ACTB (**A**) Western blotting for NUCL and ACTB, with GAPDH as the loading control. GBMc, cytosolic protein extract from GBM tissues; LGGc, cytosolic protein extract from lower grade gliomas; REFc, cytosolic protein extract from reference samples; GBMm, membrane protein extract from GBM tissues; LGGm, membrane protein extract from lower grade gliomas; REFm, membrane protein extract from reference samples. (**B**) Quantification of ACTB and NUCL protein expression in cytosolic and membrane protein fractions of pooled GBM, LGG, and reference samples showed 1.27-fold higher NUCL expression in the membrane protein extract from GBM tissues compared to the membrane protein extract from reference tissues, and almost 3-fold higher ACTB expression in the membrane protein extract from GBM *versus* the membrane protein extract from reference tissues. Band intensity of each protein was plotted after normalization to GAPDH signal of the same lane.

Western blot quantification showed that with the exception of NUCL, all of the other target proteins were over-represented in the cytosolic protein fraction of GBM tissues, compared to the reference samples (Figure 2). Western blotting of the ACTB/NUCL complex, the antigen for Nb10, showed similar expression trends for both NUCL and ACTB, with particularly lower protein expression in the GBM cytosolic protein fraction (Figure 3, GBMc) *versus* the reference cytosolic protein fraction (Figure 3, REFc), and increased protein expression in the GBM membrane protein fraction (Figure 3, GBMm) *versus* the reference membrane protein fraction (Figure 3, REFm). The ACTB/NUCL complex was validated in cytosolic and membrane protein fractions because of the reported existence of two NUCL types in GBM for cytosolic and surface occurrence [33].

**Figure 4:**
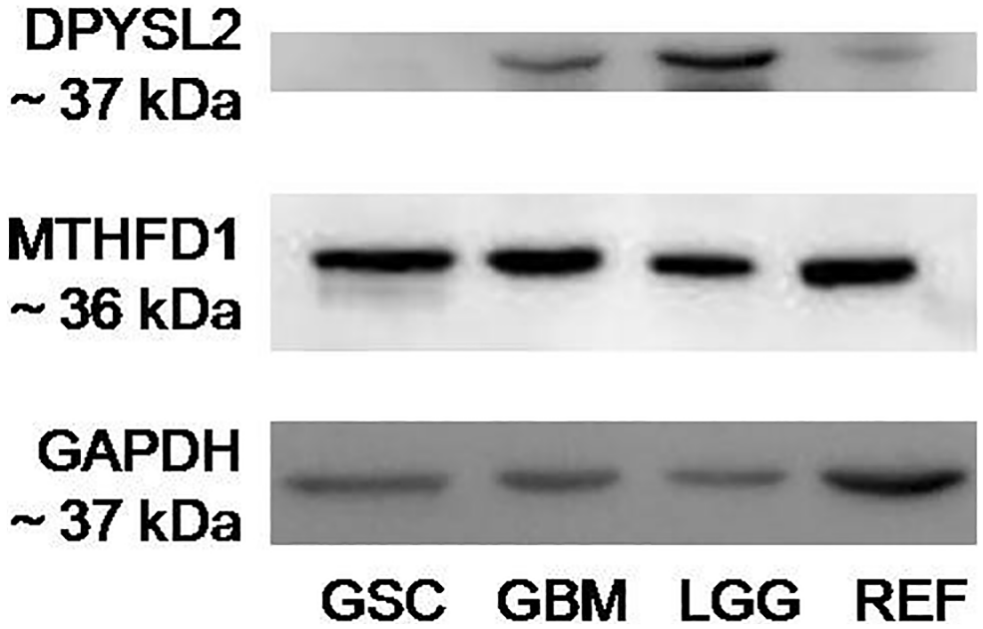
Western blotting for specific binding of Nb314 and its antigen, DPYSL2 Representative Western blotting of the two possible antigens for Nb314, including GAPDH as the loading control. GSC, protein extract from GBM stem-like cell lines after incubation with immobilized Nb314; GBM, cytosolic protein extract from GBM tissues; LGG, cytosolic protein extract from lower (Grade II and III) gliomas; REF, cytosolic protein extract from reference samples. Absence of a band with the appropriate size for DPYSL2 (~37 kDa) in the protein sample that was incubated with immobilized Nb314 prior to performing the analysis shows that Nb314 bound its antigen, which is thus no longer present in the sample, and therefore cannot be detected. Detection of the membrane probed for MTHFD1 showed bands with the appropriate size for this protein (~36 kDa) in all lanes of the analyzed membranes. The particular band in the protein samples incubated with Nb314 prior to Western blotting excludes the possibility for this protein to be an antigen for Nb314.

To determine the specific antigen for Nb314, Western blotting was performed using antigen-free protein samples (Figure 4). The absence of a band in the antigen-free protein sample (Figure 4, GSC) with the appropriate size for DPYSL2 (~37 kDa) demonstrated DPYSL2 as the antigen for Nb314. Bands with the appropriate size for MTHFD1 (~36 kDa) in all of the analyzed protein samples excluded MTHFD1 as the antigen for Nb314.

### In-silico analysis and antigen validation for class prediction

The molecular interactions database OncoFinder was used to retrieve and explore the roles of these proteins in cells, and their possible contributions to tumor formation and/or progression [34]. An interaction network was created that showed the functional links among these proteins (Supplementary Figure 1). The pathways in which these proteins are involved are given in Table 1.

**Table 1:** Interacting proteins and the pathways in which they are involved

Protein 1	Protein 2	Pathway
CRMP1	DPYSL2	CRMPs in Sema3A signaling
ACTB	ITGB3	Platelet activation
ITGB3	NUCL	Urokinase type plasminogen activator uPA and uPAR mediated signaling
CRMP1	FYN	CRMPs in Sema3A signaling
FYN	ITGAV	Signaling events mediated by focal adhesion kinase
ITGAV	NUCL	Urokinase type plasminogen activator uPA and uPAR mediated signaling
FYN	CTNNB1	E-cadherin signaling in keratinocytes
CTNNB1	VIM	WNT pathway
NUCL	NPM1	Aurora B signaling
NPM1	H2AFX	Deposition of new CENPA-containing nucleosomes at the centromere
H2AFX	TRIM28	ATM
ALYREF	SMC1A	Pre mRNA splicing
SMC1A	H2AFX	Meiotic synapsis
MTHFD1	ATIC	One carbon pool by folate
ATIC	ENTPD1	Purine metabolism
ENTPD1	POLR2E	Purine metabolism
POLR2E	ALYREF	Pre mRNA splicing

CENPA, centromere protein A

**Figure 5:**
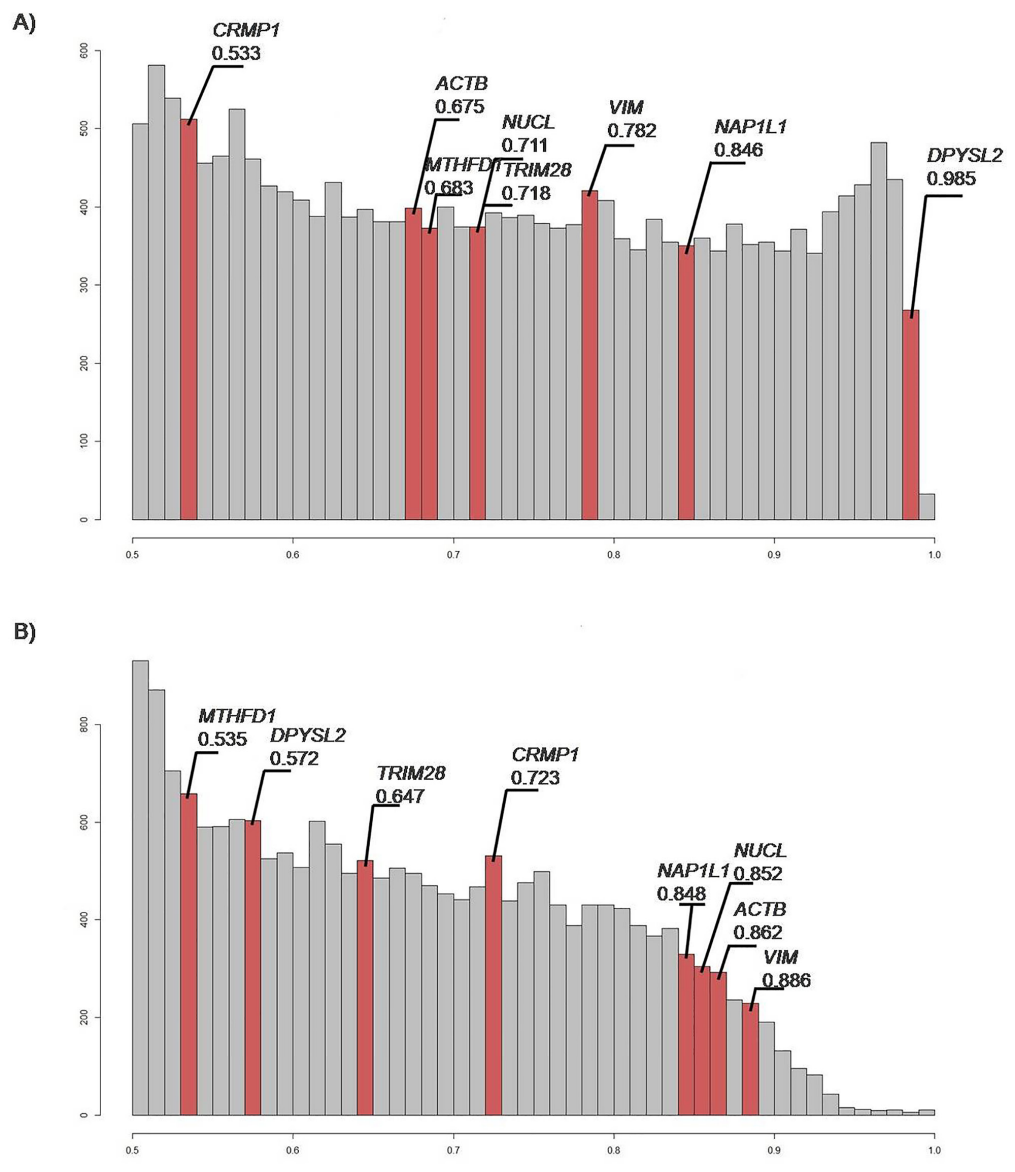
Histograms of area under curve (AUC) values AUC correlates positively with biomarker quality and varies from 0.5 to 1.0. AUC threshold for discriminating good and bad biomarkers is typically 0.7 or 0.75. Those with greater AUC are considered good-quality biomarkers, and vice-versa [87]. Red bars, genes of interest; gray bars, overall distribution. (**A**) Gene distribution analyzed, as “glioma *versus* reference”. This suggests that *DPYSL2* is a promising marker, and *NUCL*, *TRIM28*, *VIM*, and *NAP1L1* are good markers for distinguishing glioma from reference samples. (**B**) Gene distribution analyzed as “GBM *versus* LGG”. *CRMP1, NAP1L1, NUCL, ACTB* and *VIM* are indicated as genes that can be used as biomarkers to distinguish between tumor classes.

To determine whether the target genes have any value as class predictors, the mRNA expression data of these genes as retrieved from The Cancer Genome Atlas was used to calculate the area-under-the-curve (AUC) values for all of these genes in various ways. For *DPYSL2*, the “glioma *versus* reference” samples (Figure 5A) defined an AUC score of 0.985, indicating that it is a promising biomarker. Moreover, *NUCL*, *TRIM28*, *VIM*, and *NAP1L1* were indicated as good markers to distinguish glioma tissues from reference tissues (Figure 5A). For “GBM *versus* lower grade glioma (LGG)” samples, Figure 5B shows nonrandom distribution and enrichment for the *CRMP1*, *NAP1L1*, *NUCL*, *ACTB*, and *VIM* gene products with high AUC scores of 0.723, 0.848, 0.852, 0.862, and 0.886, respectively.

### Statistical analysis of gene expression in glioblastoma, lower grade glioma, and reference samples

Following OncoFinder analysis, we conducted a small scale confirmatory study on 13 GBM and 10 LGG tissue samples from Caucasian patients (Table 2) and compared them to 22 reference non-tumor brain samples. Results from our previous study, *TRIM28* and *ACTB*, were also included in the analysis [35].

**Table 2:** Clinical data for the glioma patients

Gender	Age (years)	Pathological grade	Anatomic location
M	75	IV	Occipital lobe, right
F	53	II	Parietal lobe, right
M	44	II	Insular cortex, right
M	34	IV	Frontal lobe, right
F	48	II	Insular cortex, right
M	28	II	Frontal lobe, right
M	51	II	Frontal lobe, right
M	34	III	Frontal lobe, left
M	28	III	Insular cortex, left
M	68	IV	Frontal lobe, right
F	58	IV	Temporal lobe, left
F	72	IV	Frontal lobe, left
F	50	II	Frontal lobe, left
M	45	IV	Temporal lobe, left
F	81	IV	Frontal lobe, left
M	48	IV	Temporal lobe, right
M	68	IV	Parietal-occipital lobe, left
M	25	IV	Temporal lobe, right
M	33	II	Temporal lobe, right
F	52	II	Frontal lobe, right
M	64	IV	Parietal-occipital lobe, right
M	59	IV	Parietal lobe, right
F	72	IV	Frontal lobe, left
M	52	IV	Temporal lobe, left
M	35	II	Frontal lobe, right
M	41	IV	Temporal lobe, right

**Figure 6:**
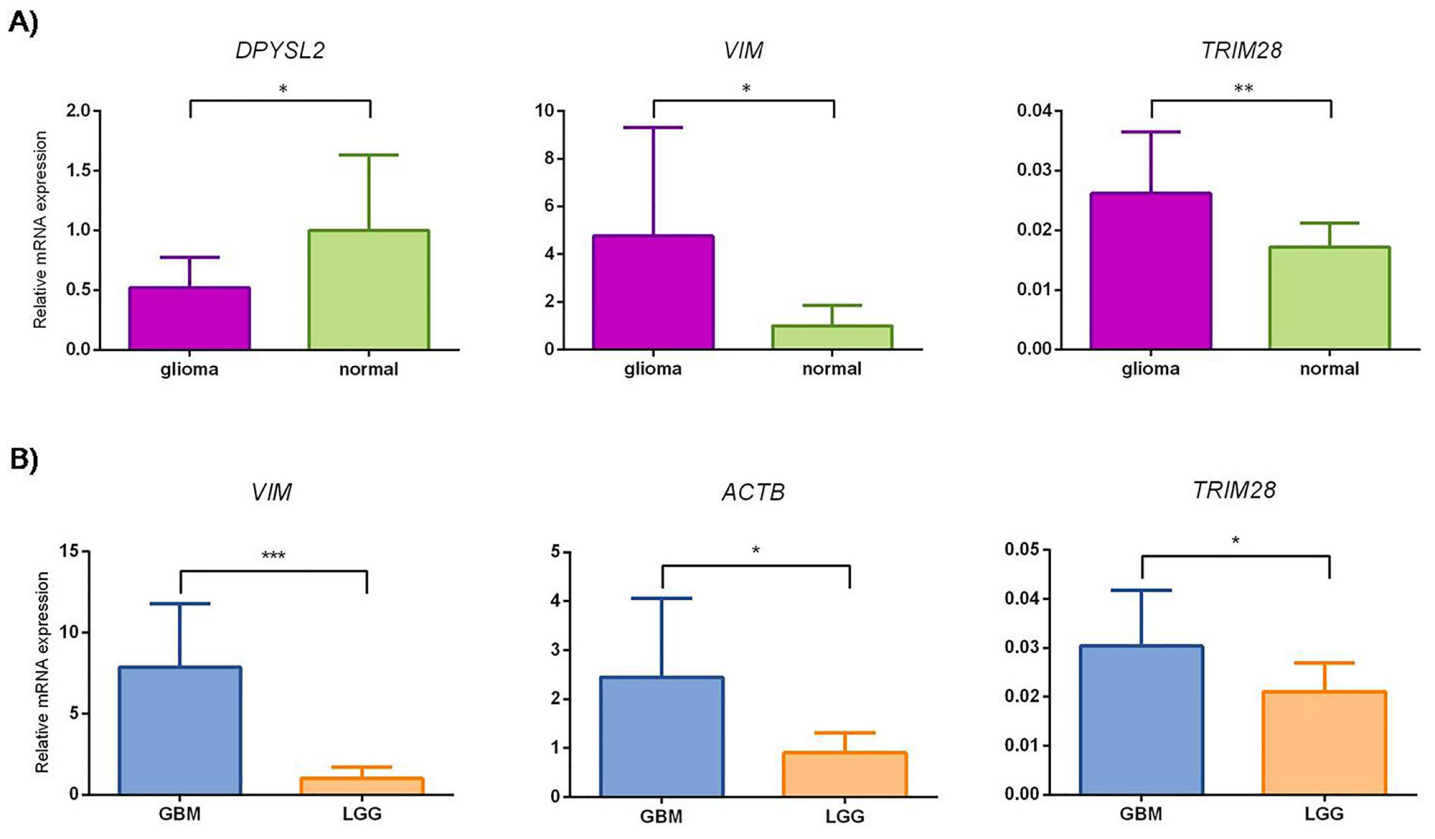
Small scale confirmatory study of relative mRNA expression levels for *DPYSL2*, *VIM*, *TRIM28* and *ACTB* The mean gene expression value corresponds to column height, with error bars representing SD. (**A**) glioma, gliomas WHO grade II, III and GBM; normal, reference samples. *, P <0.05, **, P <0.01. Mann-Whitney test showed statistically different change in expression for *DPYSL2* and *VIM* with P values 0.0158 and 0.0107, respectively. Student's *t*-test showed statistically different change in expression for *TRIM28* with P value 0.0033. (**B**) GBM, glioblastoma; LGG, lower grade gliomas. *, P <0.05, ***, P <0.001. Mann-Whitney test for *ACTB*, *TRIM28* and *VIM* showed statistically different changes in expression with P values 0.0258, 0.0237 and 0.0002, respectively.

Three genes (*TRIM28, DPYSL2* and *VIM*) were able to successfully distinguish gliomas from reference samples (Figure 6A). In addition, *ACTB*, *TRIM28* and *VIM* were able to distinguish GBM from LGG (Figure 6B). Results for genes without significant changes in expression either in “glioma *versus* reference” or in “GBM *versus* LGG” are presented in Supplementary Figure 2.

Multiple group comparisons showed strong up-regulation for *VIM, TRIM28* and *ACTB* in GBM compared to LGG and reference samples. *DPYSL2* showed slightly increased mRNA levels in the reference samples compared to both GBM and LGG groups. The other genes (i.e., *NUCL*, *NAP1L1*, *TUFM*, *CRMP1*, and *ALYREF*) did not differ significantly among these sample groups (Supplementary Figure 3).

## DISCUSSION

The aim of the present study was to increase our knowledge of the properties that distinguish GBM from LGG and from reference samples. We report a selection of nanobodies that specifically recognize GBM proteins with altered expression. Adapted expression of their target antigens in GBM samples was confirmed at mRNA and protein levels, and possible interactions among these proteins were evaluated, which suggested their involvement in different signaling pathways.

To generate an immune VHH library, initially an adult alpaca was immunized with whole GBM cells to promote the heavy-chain antibody immune response against the abundant and immunogenic GBM-specific molecules. Contrary to antigen-binding fragments of conventional antibodies, the antigen-binding fragments of heavy-chain antibodies are encoded by the single VHH gene fragments that do not get scrambled during cloning [29]. This simplifies their use, because the complete *in-vivo* matured VHH repertoire is amplified immediately in one amplicon and can be readily cloned. Then, nanobodies that recognize enriched antigens in tumor cells can be retrieved. Seven nanobodies were identified to recognize more antigen in diseased samples than in reference samples. Using a pull-down protocol, each selected nanobody captured its corresponding antigen, which was subsequently identified by mass spectrometry. With the exceptions of *DPYSL2*, *ACTB, TRIM28* and *VIM*, the genes for the antigens of the other nanobodies showed similar mRNA expression levels between diseased and healthy samples. This suggests that over-representation of the other proteins in GBM samples may have been due to post-translational modifications, epigenetic changes, or slower turn-over.

Prior to starting the bioinformatic analysis, the literature was screened to acquire information about these proteins, as given in Table 3 [36–39]. Bioinformatic analysis of genes of interest against TCGA RNA sequencing data proposed *TRIM28*, *VIM*, *NAP1L1* and *DPYSL2* as promising “glioma *versus* reference” biomarkers, and suggested *CRMP1*, *NAP1L1*, *NUCL*, *ACTB* and *VIM* for discrimination between GBM and LGG. These results were confirmed by analyzing the relative mRNA expression levels of the genes of interest on a small patient cohort. Our confirmatory qPCR study showed differential gene expression levels for *DPYSL2*, *VIM* and *TRIM28* in “glioma *versus* reference” samples. Moreover, from the suggested biomarkers to distinguish between GBM and LGG, qPCR confirmed the potential roles of *VIM*, *TRIM28* and *ACTB* as markers for class differentiation.

**Table 3:** Antigen roles, as indicated from the literature

Antigen	Role	Literature
NUCL	Abundant in exponentially growing cells Cytoplasmic and surface NUCL in gliomas Surface NUCL is dependent on association with actin cytoskeleton Cell-surface NUCL is a novel angiogenesis marker	[33, 68–70]
VIM	Role in formation of lamellipodia and invadipodia during cell invasion and migration Typical for infiltrative gliomas with poor prognosis Marker for epithelial to mesenchymal transition Differentially expressed in glioblastomas	[39, 71–73]
NAP1L1	Human NAP1L1 locates within the nucleus of dividing cells Co-existence of NAP1L1 and NAP1L4 in neural stem cells and neurons Transcriptional regulation *NAP1L1* is overexpressed in small-intestinal carcinoids	[74–78]
TUFM	Chaperone-like properties in protein folding and renaturation under stress conditions Translational expression of mitochondrial DNA Involved in head and neck squamous cell carcinoma Overexpressed in colorectal carcinoma	[79–81]
DPYSL2	Highly expressed during brain development, rarely in post-developmental brain Over-expressed in colorectal carcinoma Altered expression in glioblastomas Promoter of microtubule assembly and neuronal development	[38, 71, 82, 83]]
CRMP1	Decreased in glioblastomas expressing EGFRvIII Invasion suppressor gene in lung cancer Associated with microtubule-containing structures in mitotic cells	[38, 84, 85]
ALYREF	Transport of fully processed mature mRNA out of the nucleus NUP107 complex (consisting of ALYREF, SNRPE, NUP107 and NUP50) is altered in 19% of glioblastomas	[37, 86]

Our network implies that these analyzed proteins are involved in alterations in normal cell metabolism using signaling pathways, including the WNT and ATM pathways, as well as Aurora B, Sema3A, and E-cadherin signaling. Overexpression of Aurora B mRNA and protein has been detected in a number of cancers [40–42], and tumorigenic changes have also been observed for the WNT and ATM pathways [43–45]. This suggests that GBM does not evolve through a single pathway, but through numerous pathways, which will be related to angiogenesis, and cell invasion, signal transduction, and metabolism [46, 47]. This complex nature of GBM thus implies that successful therapies will need to target different biological processes, to parallel these diverse pathways that GBM uses to ensure growth [48].

## CONCLUSIONS

With combined use of nanobodies, proteomics and bioinformatics, we identified a set of proteins with altered expression in GBM samples. The analysis of the genes of interest using RNA sequencing data from TCGA suggested the possible use of some of them as biomarkers for glioma class differentiation, with *DPYSL2* demonstrated as a glioma-specific biomarker. The use of *CRMP1*, *NAP1L1*, *NUCL*, *ACTB*, and *VIM* as potential class predictors also needs to be validated in biological fluids. Our bioinformatic results were verified using a small scale confirmatory study. Validation of our findings on a dataset that contains a greater number of glioma samples and reference samples should now be initiated. Further studying the significance of these biomarkers for glioma formation, progression and prognosis would help development of new treatment strategies, which may be beneficial for GBM patients.

## MATERIALS AND METHODS

### Ethics statement

The present study was approved by the National Medical Ethics Committee of the Republic of Slovenia (Numbers: 92/06/12, 89/04/13 and 95/09/15). Written informed consent was signed by the patients prior to their surgery. Reference samples were obtained during autopsies, following the legal regulations valid for the Republic of Slovenia. All of the samples used in this study are anonymous.

### Preparation of protein extracts

Protein extracts used for phage particle enrichment and during panning were obtained from two commercially available GBM stem-like cell lines: NCH644 and NCH421K cells (Cell Line Service, Eppelheim, Germany) [49, 50]. Cells were grown in complete medium, as neurobasal medium (Gibco) plus 20 μM L-glutamine, 100 U/mL penicillin, 100 μg/mL streptomycin, 2% B-27 supplement, 20 ng/mL basic fibroblast growth factor, 20 ng/mL epidermal growth factor, and 1 U/mL heparin.

Brain tissues originated from the hippocampus and subventricular and periventricular zones of 10 patients, were dissected out during autopsies and used as reference samples. Tissue samples were sealed in containers, labeled, snap frozen, and kept at -80 °C until they were used for protein extraction.

Glioblastoma samples from 13 patients (nine male, four female; aged 25-81 years), and lower grade glioma samples (WHO grade II and III) from 10 patients (six male, four female; aged 28-53) were dissected out during surgery, sealed in sterile containers, labeled, snap frozen, and kept at -80 °C until they were used for protein extraction. Data is presented in Table 2.

Two commercially available kits were used to separate the membrane and cytosolic protein fractions from all of the samples: ProteoExtract Transmembrane Protein Extraction kit (Novagen), and ProteoExtract Native Membrane Protein Extraction kit (Calbiochem). Protein concentrations were determined using the Bradford method [51].

### Bio-selection and ELISA screening

An existing nanobody library was used in the present study. An adult alpaca was immunized with whole GBM cells, as indicated above and described previously [35]. The nanobody library was constructed by the Nanobody Service Facility (Vrije Universiteit Brussel, Brussels, Belgium), according to their established protocols [52–54].

Phage enrichment (i.e., bio-selection) was performed on solid-phase coated cytosolic proteins isolated from NCH644 and NCH421K GBM stem-like cell lines. Protein for coating of the GBM and reference samples was at 200 μg/mL (100 μL/well). Three rounds of phage enrichment were performed, following a published protocol [53]. Briefly, phages were incubated with the protein samples coated on plastic microtiter plates, extensively washed and target-specific phages eluted with triethylamine. Eluted phages were used to infect fresh *Escherichia coli* cells for overnight proliferation in 2× Yeast Extract and Tryptone with 100 μg/mL ampicillin and 70 μg/mL kanamycin. The following morning, phage particles were precipitated with PEG-6000/ 2.5 M NaCl and used in the next round of panning. Periplasmic extract ELISA was used to screen for clones expressing nanobodies with specificity for GBM proteins [53], with protein for ELISA at 2 μg/mL (100 μL/well) for GBM and reference samples. Briefly, individual clones were inoculated in 1 mL Terrific Broth medium and the nanobody expression was induced by adding 10 μL 1 M isopropyl β-D-1-thiogalactopyranoside. The next day, periplasmic extracts were obtained by incubating the *E. coli* initially in a hypertonic Tris/ EDTA/ sucrose solution, and then in a hypotonic solution, following the addition of distilled H_2_O. These periplasmic extracts were applied in parallel to wells that were coated overnight with proteins from GBM and reference samples. To detect the antigen-specific nanobodies, 100 μL/well primary and secondary antibodies, mouse anti-hemagglutinin, and goat-anti-mouse IgG (whole molecule)–alkaline phosphatase conjugate (both Sigma Aldrich), respectively, were used. The ELISA signals were measured at 405 nm after applying 100 μL/well alkaline phosphatase conjugate (Sigma Aldrich). The nanobody genes for the ELISA positive clones were amplified using colony PCR (6 min at 95 °C, 45 s at 94 °C, 45 s at 55 °C, 45 s at 72 °C, 10 min at 72 °C) with the primers RP (5'→3' TCA CAC AGG AAA CAG CTA TGA C) and GIII (5'→3' CCA CAG ACA GCC CTC ATA G), and were sequenced at Macrogen (Amsterdam, The Netherlands). Those that had appropriate sequences were chosen for large-scale expression.

### Recloning and nanobody production

The nanobody genes were recloned in the pHEN6c vector and transformed into *E. coli* WK6 cells prior to large-scale production. The nanobodies were produced as described previously [55, 56]. Briefly, clones were grown overnight in Luria–Bertani–Miller medium. These cultures were used to inoculate 1.5 L Terrific Broth medium for each clone. Nanobody production was induced by adding 1.5 mL 1 M isopropyl β-D-1-thiogalactopyranoside, and the cultures were further shaken overnight. The periplasmic extracts were incubated with Ni^+^-nitrilotriacetic acid agarose (Qiagen), overnight at 4 °C. Immobilized nanobodies were washed with three column volumes phosphate-buffered saline (PBS) and then eluted with one column volume freshly prepared PBS supplemented with 0.5 M imidazole. Nanobodies were further purified by size-exclusion chromatography.

### Antigen identification

**Figure 7:**
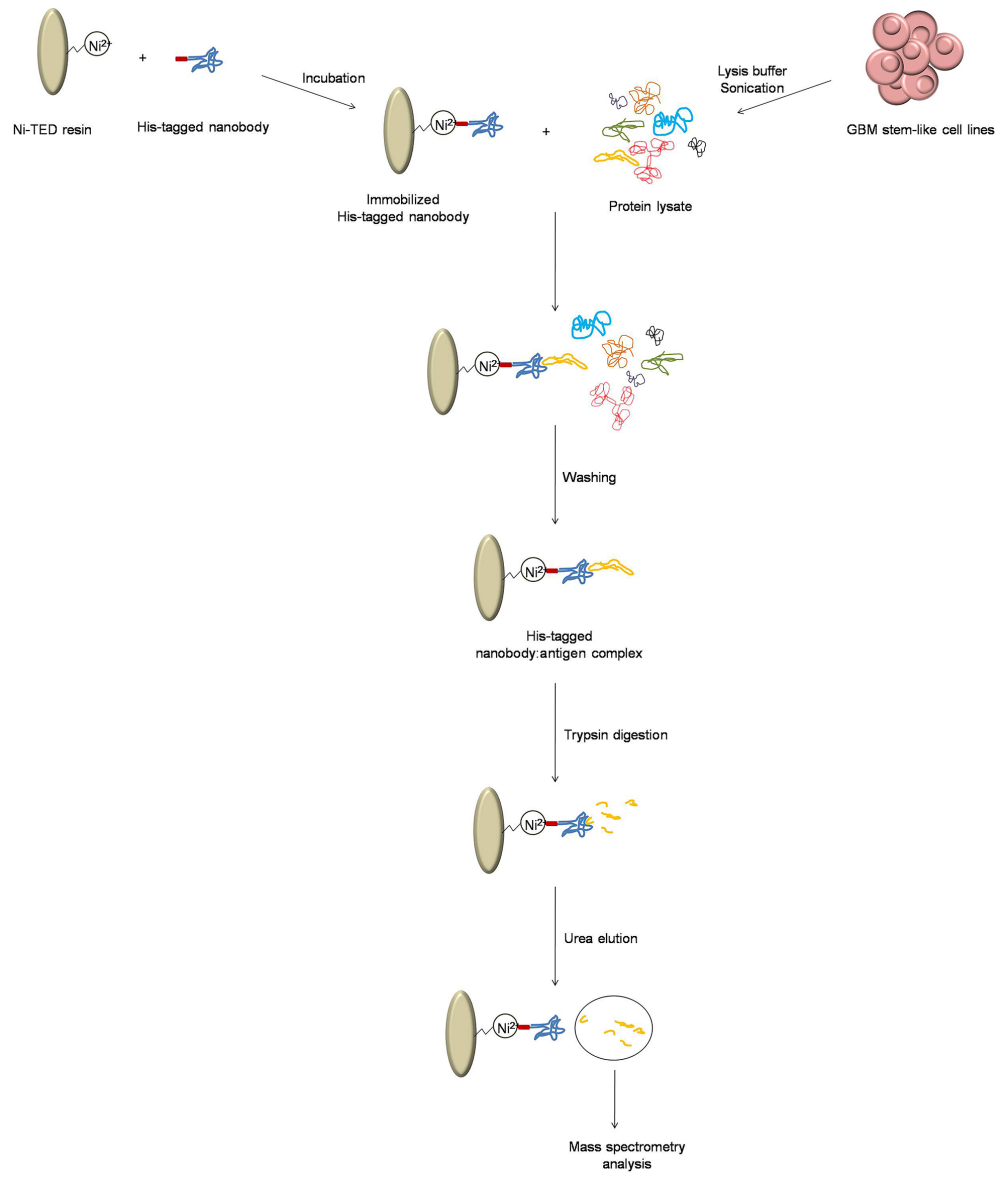
Schematic representation of the sample preparation for mass spectrometry The process to obtain nanobody: antigen pairs, and antigen preparation for mass spectrometry analysis.

Protein lysates from NCH644 and NCH421K cells were freshly prepared as follows: ~60 ×10^6^ GBM cells were centrifuged at 125x *g* for 5 min at room temperature, supernatant was removed, and the cells were resuspended in lysis buffer (PBS supplemented with 0.1% NP-40). Cells were sonicated for 1 min (30 ms ON, 30 ms OFF). The resulting suspension was centrifuged at 290x *g* for 5 min at 4 °C, and the resulting supernatant was used for the antigen capture (Figure 7). Nanobodies were immobilized on Ni-(tris(carboxymethyl)ethylene diamine) resin (Machery-Nagel), and then incubated on a rotating platform for 1 h at 4 °C with fresh protein lysate. Supernatant was then removed, and the samples were washed three times with equilibration buffer (50 mM NaH_2_PO_4_·2H_2_O, 300 mM NaCl) and transferred to new microcentrifuge tubes. Samples were then washed five times with PBS, and the protein was digested with 10 μL 5 ng/μL trypsin solution (0.5 μg porcine trypsin in 100 μL PBS), overnight at room temperature. The next day, supernatant was removed, samples were washed twice with PBS, and the antigens were separated from the nanobodies with 150 μL 6 M urea. Urea elutions and overnight digests were cleaned up using stop-and-go-extraction tips (Stage Tips) [57], and analyzed using liquid chromatography–tandem mass spectrometry with the Easy-nLC system connected to an electron-transfer dissociation ion trap (Amazon; Bruker). The chromatogaphy was developed using a 75-min discontinuous gradient from 0% to 80% methanol in 0.1% formic acid. The tandem mass spectrometry spectra were searched against the human database using the X!tandem and MASCOT search engines, allowing a 5% false-recovery rate.

### Western blotting

The vast majority of nanobodies bind to conformational epitopes, which are lost in denatured proteins [58]. This complicates the use of nanobodies as detection probes in Western blotting. Therefore, standard monoclonal antibodies (Sigma Aldrich) were used in the Western blots to confirm the differential occurrence of the target proteins. Protein extracts from glioma samples were divided into two groups: GBM, as 13 pooled tissue samples from patients with WHO Grade IV gliomas; and LGG, as 10 pooled tissue samples from patients with WHO Grade II and Grade III astrocytomas. Pooled protein extracts from 22 *post-mortem* brain samples were used as the reference samples. Here, ~20 μg to 30 μg pooled protein extracts were separated using 4% to 12% NuPAGE Bis-Tris Mini Gels (Invitrogen), and transferred to an Immobilion-P Transfer Membrane (Milipore). BlueStar Prestained Protein Ladder (Nippon Genetics) was used as the protein marker. Residual protein binding sites on the membranes were blocked with 5% PBS–milk for 1 h at room temperature. A mouse monoclonal anti-GAPDH antibody (Sigma Aldrich) was used for the loading control. The incubations with the primary antibodies were at 4 °C overnight, with shaking at 60 rpm. The incubations with the secondary anti-mouse IgG (whole molecule)–peroxidase antibody produced in goat (Sigma Aldrich) were for 1 h at 4 °C, with shaking at 60 rpm. The bands were revealed with SuperSignal West Pico Chemiluminescent Substrate (Thermo Scientific), visualized with Fujifilm LAS-4000 (Tokyo), and analyzed with the Multi Gauge version 3.2 software. The numerical values of the antigen intensities were calculated as the ratio between the arbitrary units (AU) of the antigen and the loading control, as GAPDH (AU(antigen)AU(GAPDH)).

**Figure 8:**
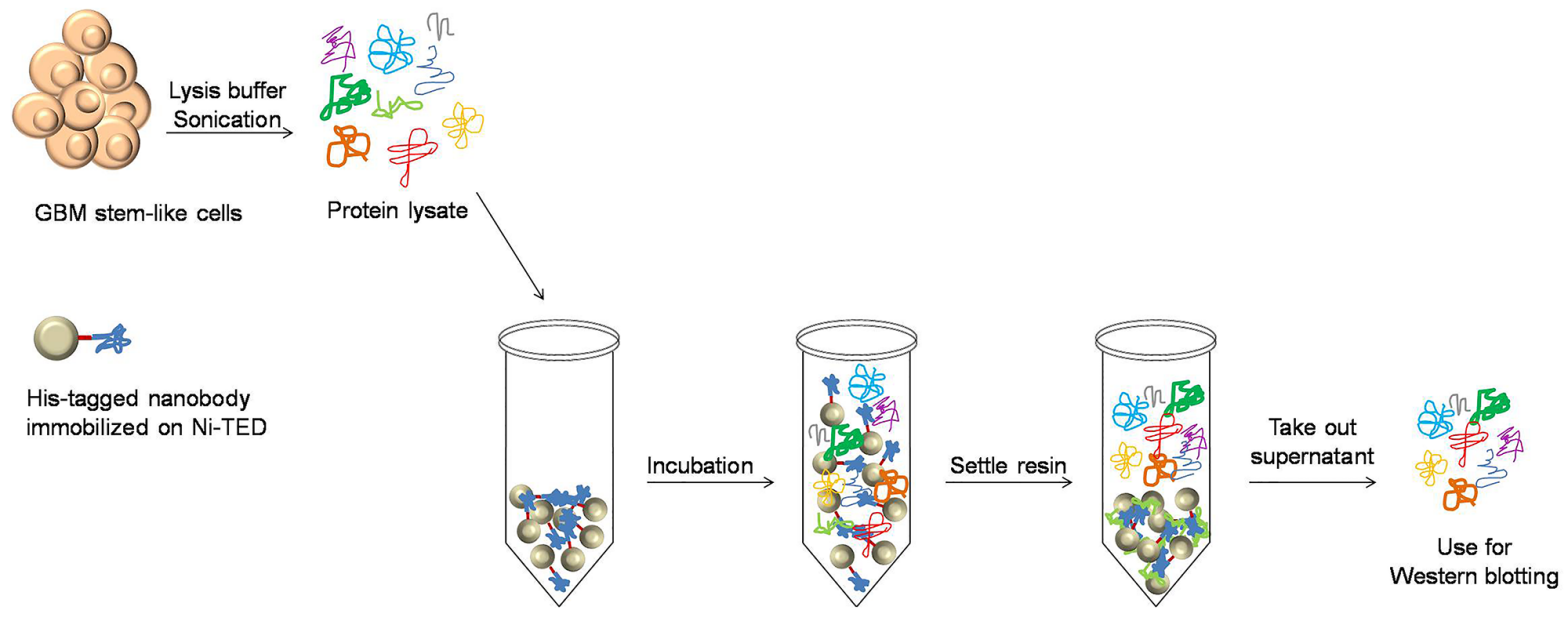
Protein sample preparation for determination of the Nb314 antigen Preparation of antigen-free protein lysate used to determine the matching antigen for Nb314.

To determine the specific antigen for Nb314 (Figure 8), this nanobody was immobilized on Ni-(tris(carboxymethyl)ethylene diamine) resin (Machery-Nagel) and incubated with fresh protein lysate (for preparation see “Antigen identification”) on a rotating platform for 1 h at 4 °C. After this incubation, the resin was allowed to settle and the supernatant (i.e., antigen-free protein lysate) was transferred to a new microcentrifuge tube. Total protein concentration was determined using the Bradford method. Here, ~20 μg to 30 μg protein extracts (antigen-free protein lysate, protein lysates from GBM, LGG and reference samples) were used for Western blotting. One membrane was probed with mouse monoclonal anti-DPYSL2 monoclonal antibody (Sigma Aldrich), and another with the MTHFD2 monoclonal antibody (M01), clone 4G7-2G3 (Abnova). Probing with primary and secondary antibodies, band detection and quantification were performed as described above.

### Bioinformatics

Possible interactions between the identified proteins were analyzed using the OncoFinder software [59] and the Reactome [60], Kyoto Encyclopedia of Genes and Genomes [61], and HumanCyc [(www.humancyc.org)] molecular interactions databases. The results from our previous study for TRIM28 and ACTB [35] were also included in the present analysis. Furthermore, the genes of interest were analyzed against TCGA RNA sequencing dataset which consists of 171 GBM, 530 LGG and five reference samples. This contains information about 19801 genes, including the genes of interest in the present study (i.e., *CRMP1*, *DPYSL2*, *NAP1L1*, *NUCL*, *TRIM28*, *VIM*, and *ACTB*), with the sole exception of *ALYREF*. The genes were checked for specific overexpression or underexpression among the data available from TCGA, using “cancer-to-normal” ratios calculated with OncoFinder. The AUC values for all of these genes were calculated as “GBM *versus* LGG”, “GBM *versus* LGG *versus* reference” and “glioma *versus* reference”. The RNA sequencing data were normalized using the DeSeq2 package, and the Firehose software was used to retrieve data.

### Quantitative reverse transcription polymerase chain reaction

qPCR was used to determine changes in mRNA expression in the samples. mRNA was extracted from 13 GBM, 10 LGG and 22 paired reference samples. Extractions were performed with TRI Reagent (Sigma Aldrich), according to the manufacturer instructions. mRNA concentrations were estimated using a NanoDrop ND-1000 (NanoDrop Technologies, USA), and mRNA purities were determined using A_260_/A_280_ and A_260_/A_230_ ratios. The RNA integrity numbers (RINs) were determined using a bioanalyzer (Agilent 2100; Agilent Technologies, USA). Twelve samples with insufficient RNA quality (two GBM, one LGG, nine reference samples, with RINs from 2.2-2.9) were excluded from further analysis. Then, 2 μg of each mRNA sample was used for cDNA reverse transcription. The samples were treated with recombinant RNase-free DNase I (Roche) to remove possible DNA traces (15 min 30 °C, 10 min 75 °C) and transcribed with Transcriptor Universal cDNA Master (Roche), as follows: 5 min at 25 °C, 10 min at 55 °C, and 5 min at 85 °C.

The qPCR was performed using a Roche LightCycler 480 platform. The total reaction volume was 5 μL, and consisted of: 0.75 μL cDNA, 2.5 μL 2× LightCycler 480 SYBR Green I Master (Roche), 0.3 μL of each of the 2.5 mM primers, and 1.15 μL distilled H_2_O. The reactions were performed in triplicate using the following thermal cycling: pre-incubation 10 s at 95 °C; cycling, 20 s at 60 °C, 20 s at 72 °C, for 45 cycles; melting curve, 5 s at 95 °C, 1 min at 65 °C; continuous at 97 °C; and cooling, 30 s at 4 °C. From the five candidate reference genes (i.e. *TBP*, *HPRT1*, *RPL13A*, *GAPDH*, *CYC1*) chosen from the literature [62–64], *RPL13A* and *CYC1* were selected for gene normalization using the NormFinder algorithm [65]. The primers for the reference genes (Table 4) were chosen from the literature [64], while the primers for the genes of interest (Table 5) were obtained from the PrimerBank PCR primer database for quantitative gene expression analysis (https://pga.mgh.harvard.edu/primerbank/) [66]. Relative quantification was performed as previously described [67]. For samples following Gaussian distribution differential expression analyzed as “GBM *versus* LGG *versus* reference” was calculated using one-way ANOVA and Holm-Sidak's corrections for multiple comparisons. For samples not following Gaussian distribution differential expression was calculated using Kruskal-Wallis test. In both cases P ≤0.05 was considered statistically significant (*, P ≤0.05; **, P <0.01; ***, P <0.001; ****, P <0.0001).

**Table 4:** Primers for candidate reference genes

Gene	Primer sequence (5'→3')	Ampliconlength (bp)	Gene name	Function
TBP	F: CAG CAT CAC TGT TTC TTG GCG TR: AGA TAG GGA TTC CGG GAG TCA T	232	TATA-box binding protein	General transcription factor
HPRT1	F: CAG CCC TGG CGT CGT GAT TAG TR: CCA GCA GGT CAG CAA AGA AT	226	Hypoxanthine-guanine phosphoribosyltransferase	Metabolic salvage of purines
RPL13A	F: CCT GGA GGA GAA GAG GAA AGA GAR: TTG AGG ACC TCT GTG TAT TTG TCA A	126	60s ribosomal protein L13a	Component of 60Sribosomal unit
GAPDH	F: TCG CCA GCC GAG CCA CAT CR: CGT TCT CAG CCT TGA CGG TGC	222	Glyceraldehyde-3-phosphatedehydrogenase	Glycolysis enzyme
CYC1	F: GAG GTG GAG GTT CAA GAC GGR: TAG CTC GCA CGA TGT AGC TG	160	Cytochrome C-1	Mitochondrial electron transport

F, forward; R, reverse

**Table 5:** Primers for the genes of interest

Gene	Primer sequence (5'®3')	Amplicon length (bp)
NUCL	F: GAA CCG ACT ACG GCT TTC AATR: AGC AAA AAC ATC GCT GAT ACC A	93
ACTB	F: CCA ACC GCG AGA AGA TGAR: CCA GAG GCG TAC AGG GAT AG	97
VIM	F: TGC CGT TGA AGC TGC TAA CTAR: CCA GAG GGA GTG AAT CCA GAT TA	248
NAP1L1	F: AAA GCA CGT CAG CTA ACT GTTR: TTG AGA GCA TTC ACT CGT CTT TT	146
TUFM	F: AAA GAA GGG AGA CGA GTG TGAR: TGT GGA ACA TCT CAA TGC CTG	80
DPYSL2	F: GTG ACT ACT CTC TGC ATG TGG AR: TTA CCC CGT GAT CCT TCA CAA	87
CRMP1	F: AGT GAC CGA CTC CTC ATC AAAR: CCA GGA ACG ATT AAG TTC TCT CC	119
ALYREF	F: ACA TTC AGC TTG TCA CGT CACR: TCT AGT CAT GCC ACC TCT GTT TA	77
TRIM28	F: TGA GAC CTG TGT AGA GGC GR: CGT TCA CCA TCC CGA GAC TT	93

F, forward; R, reverse

### Statistical analysis

To confirm the bioinformatic findings, relative mRNA expression levels of the genes of interest were analyzed as “glioma *versus* reference” and “GBM *versus* LGG” in GraphPad Prism 6 (GraphPad Software Inc., La Jolla, CA, USA). For samples following Gaussian distribution analysis was performed using unpaired two-tailed Student's *t*-test. Mann-Whitney test was used where samples did not follow Gaussian distribution. In both cases P ≤0.05 was considered statistically significant (*, P ≤0.05; **, P <0.01; ***, P <0.001; ****, P <0.0001).

## SUPPLEMENTARY MATERIALS FIGURES


