# Supplementary material for: Differentially expressed proteins in glioblastoma multiforme identified with a nanobody-based anti-proteome approach and confirmed by OncoFinder as possible tumor-class predictive biomarker candidates
Source: Oncotarget. 2017 Apr 24;8(27):44141–58. doi: 10.18632/oncotarget.17390 (PMC5546469; doi:10.18632/oncotarget.17390)
Supplement: Supplementary file 1 [file oncotarget-08-44141-s001.pdf]

Differentially expressed proteins in glioblastoma multiforme identified with a nanobody-based anti-proteome approach and confirmed by OncoFinder as possible tumor-class predictive biomarker candidates

SUPPLEMENTARY MATERIALS

SUPPLEMENTARY FIGURES

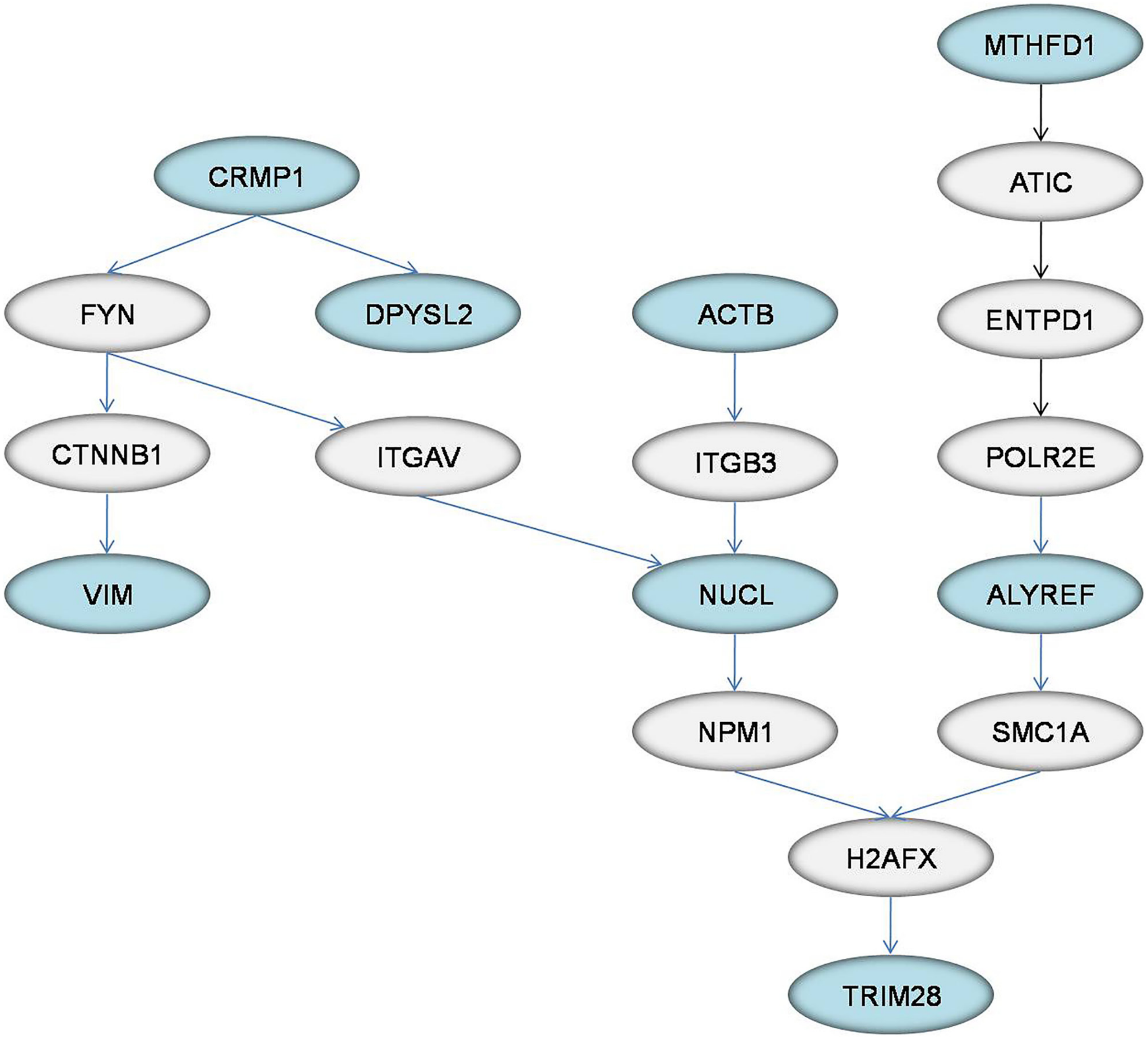

Supplementary Figure 1: Interaction map of the top differentially expressed proteins identified using nanobodies. Correlations among the proteins studied with OncoFinder. Blue, proteins investigated in this study; gray, other proteins involved in the interaction network; black arrows, interactions through intermediate compounds; blue arrows, direct activation of Protein 2 (protein at the end of the arrow) by Protein 1 (protein at the beginning of the arrow). NAP1L1 and TUFM were not identified as part of the network, as there is relatively little information available about their possible interactions.

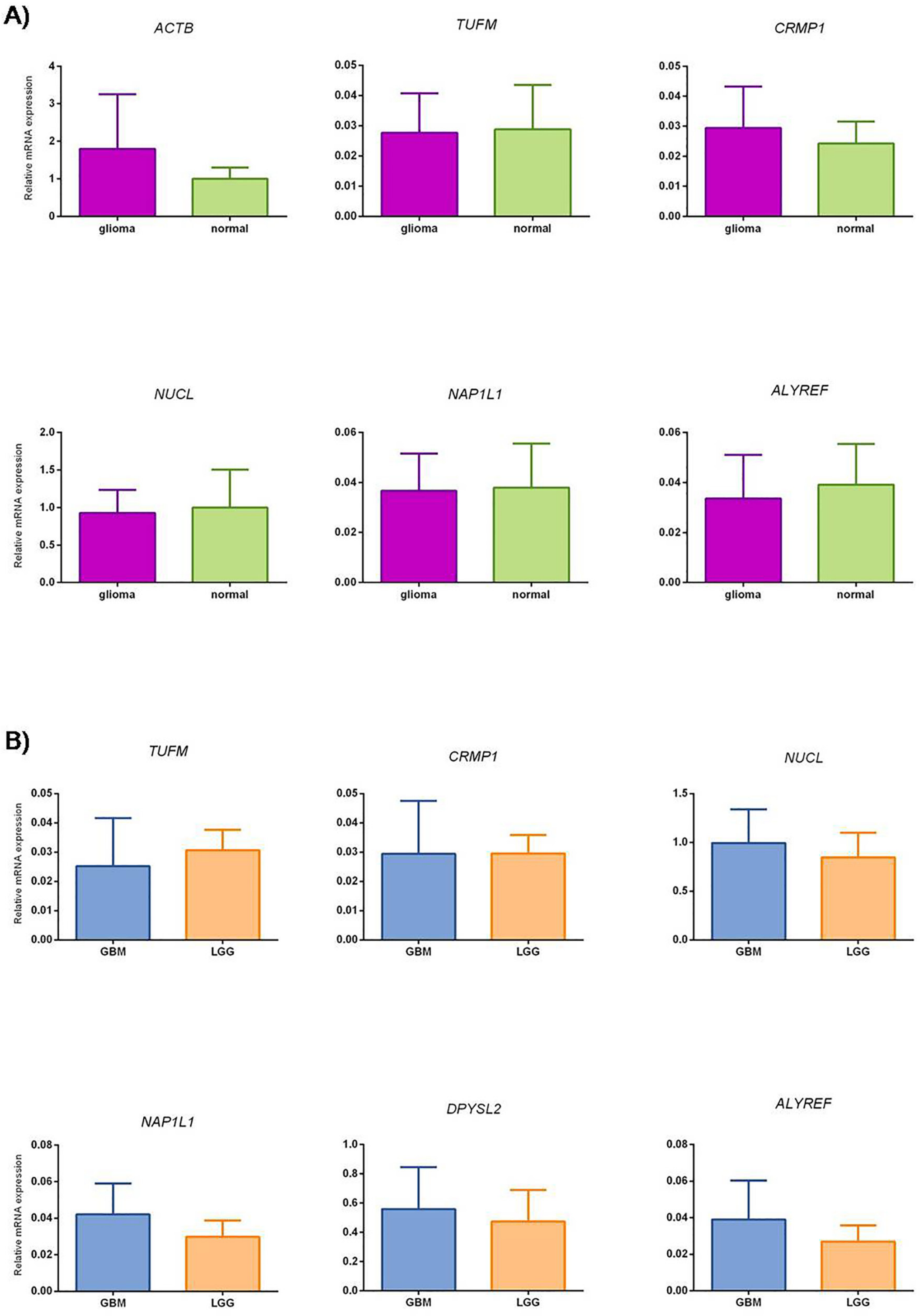

Supplementary Figure 2: Small scale confirmatory study of relative mRNA expression levels for selected genes of interest. Data are mean gene expression ±SD. All samples present with Gaussian distribution. **(A)** glioma, lower grade gliomas (WHO grade II, III) and GBM; normal, reference samples. Student's *t*-test did not show statistically different changes in expression. **(B)** GBM, glioblastoma; LGG, lower grade gliomas. Student's *t*-test did not show statistically different changes in expression.

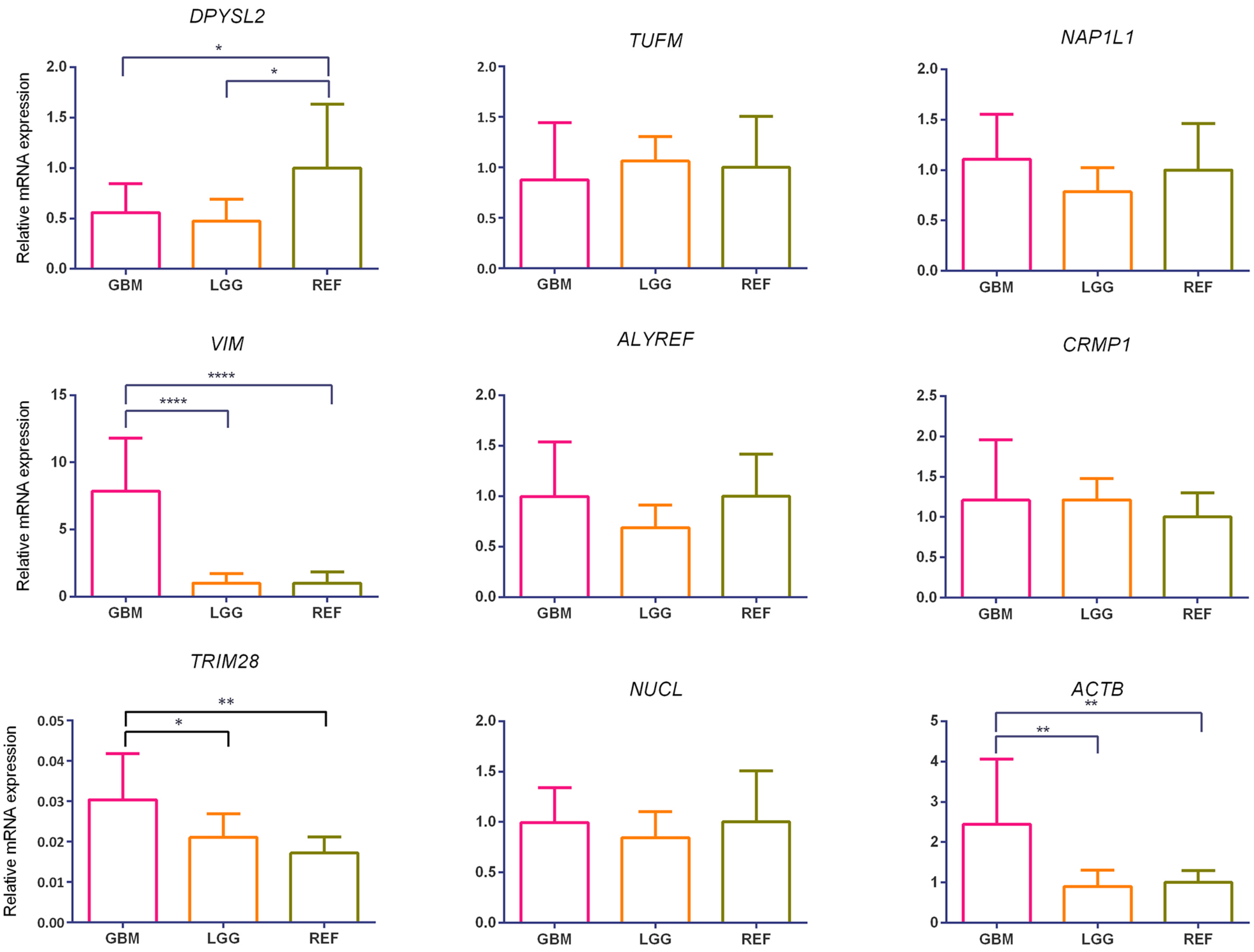

Supplementary Figure 3: Relative mRNA expression levels for selected genes of interest. The mean gene expression value corresponds to column height, with error bars representing SD. GBM, glioblastoma; LGG, lower grade gliomas; REF, reference samples. *, P <0.05, **, P <0.01; ****, P <0.0001.
